# PD08 Clinical And Cost Effectiveness Of Selected Diabetes Medicines In Ghana - An Adaptive Health Technology Assessment

**DOI:** 10.1017/S0266462324002770

**Published:** 2025-01-07

**Authors:** Hannah Asante, Richmond Owusu, Brian Asare, Ivy Amankwah, Sergio Torres Reuda, Joseph Kazibwe, Francis Ruiz

## Abstract

**Introduction:**

There is a need to consider whether treatments included in essential medicine lists, standard treatment guidelines, and health benefits packages are cost effective, improve financial sustainability, and increase equitable access to health care. This assessment evaluated the cost effectiveness of selected antidiabetic medicines for inclusion on Ghana’s Essential Medicines List and updated standard treatment guidelines, and reimbursement by the National Health Insurance Authority.

**Methods:**

This study was produced in line with the broad steps of the Ghana health technology assessment (HTA) process guideline using an adaptive HTA (aHTA) approach and following the process used by the National Cancer Grid of India. High quality HTA evidence was sourced from four HTA agencies based on the population (children aged two years or older and adults older than 18 years using antidiabetics), intervention, comparator, and outcomes framework using an adaptability checklist developed by the researchers. A price benchmarking analysis was conducted to generate context relevant evidence on medicine prices in terms of local value for money.

**Results:**

The study found that all medicines evaluated (sitagliptin, vildagliptin, saxagliptin, insulin detemir, insulin degludec, insulin glargine, insulin glusiline, insulin aspart, and insulin lispro) were efficacious. The price benchmark analysis showed that insulin detemir, glargine, and degludec had higher price ratios than their comparators, and an annual drug cost per patient that was approximately two to four times higher. Insulin lispro, aspart, and glulisine had price ratios of 0.22 to 0.44 and an estimated annual cost of GHS1,894 to GHS3,552 (USD163.3 to USD306.2), which was two to five times higher per patient than the comparators. The cost of saxagliptin and vildagliptin were four and three times lower than those in the benchmark country (the UK).

**Conclusions:**

The study revealed that all medicines included are efficacious and potentially cost effective. The price benchmark analysis showed that, except for gliclazide 80 mg, Ghana is paying less for antidiabetic medications than the UK. Cost effectiveness may not be a sufficient basis to include or exclude medicines for reimbursement because they have a potentially significant budget impact for the payer.

